# Dissolution Effect of Alteplase on Arterial Blood Clot Model of Hypertensive Intracerebral Hemorrhage Patients *in vitro*

**DOI:** 10.3389/fneur.2022.729727

**Published:** 2022-02-18

**Authors:** Xiaoming Jiang, Yongyu Lu, Xiaogang Hu, Xiaochun She

**Affiliations:** ^1^Department of Neurosurgery, Rudong Hospital Affiliated to Nantong University, Nantong, China; ^2^Department of Neurosurgery, Haimen District People's Hospital, Nantong, China; ^3^Department of Respiratory Medicine, Rudong Hospital Affiliated to Nantong University, Nantong, China

**Keywords:** alteplase, urokinase, hypertensive cerebral hemorrhage, arterial blood, dissolution

## Abstract

**Objective:**

To explore the dissolution effect of alteplase (rt-PA) on arterial blood clots of patients with hypertensive cerebral hemorrhage *in vitro* and analyze the optimal concentration and action time of rt-PA for intracranial hematomas.

**Methods:**

The arterial blood of 35 patients with confirmed hypertensive cerebral hemorrhage were collected, centrifuged, and the serum was aspirated to prepare the blood clot model. The 0.125, 0.25, 0.5, 1, 2, and 3 mg t-PA, 20,000 U, and 40,000 U urokinase (u-PA) were taken for the corresponding blood clot for dissolution test. The blood clot volume and dissolution volume was measured at 0, 30, 60, 90, 120, and 150 min.

**Results:**

Without intervention, the blood clot volume of men was higher than that of women at 0, 30, 60, and 90 min (*P* < 0.05). Without intervention, hematocrit (HCT) was correlated with blood clot volume and the correlation decreased with time. The 30, 60, and 90 min dissolution curves of each group showed an upward trend (*P* < 0.05), and the dissolution curves tended to be flat at 120 min and 150 min. The dissolution volume of.125 mg/3 ml, 0.25 mg/3 ml, 0.5 mg/3 ml rt-PA, 20,000 U, 40,000 U u-PA was higher than that of 1, 2, 3 mg/ml rt-PA (*P* < 0.05). The dissolution volume of.125 mg/3 ml, 0.25 mg/3 ml, 0.5 mg/3 ml rt-PA was not significantly different from 20,000 and 40,000 U u-PA (*P* > 0.05). Gender differences did not affect the effects of the above drugs.

**Conclusion:**

*In vitro*, low-concentration rt-PA has a better dissolution effect, and it shows a time-dependent effect, reaching the highest effect in 90 min.

## Introduction

Hypertensive intracerebral hemorrhage has a high incidence, a long treatment period, and a poor prognosis, which causes a large burden on the family and society (1–3). At present, traditional craniotomy is traumatic and causes inevitable damage to normal brain tissue, and the prognosis does not show obvious advantages over conservative treatment (4, 5). Phase II and Phase III clinical trials of minimally invasive surgery plus alteplase (rt-PA) for intracerebral hemorrhage evacuation (MISTIE) confirmed that minimally invasive puncture and drainage of the hematoma and injection of rt-PA into the hematoma could effectively relieve the hematoma occupying effect and secondary damage (5).

The main component of rt-PA is glycoprotein which can bind to fibrin through lysine residues and activate plasminogen bound to fibrin to turn it into plasmin, thereby dissolving blood clots or thrombus. Rt -PA is a third-generation thrombolytic drug with the characteristics of safety, efficiency, and convenience (6). However, studies have observed that rt-PA had dose-related neurotoxicity and overdose could cause edema around the lesion and destroy the blood-brain barrier, which was not conducive to the prognosis (7–12). Therefore, the drug concentration and action time of rt-PA used in the treatment of intracranial hematoma have become a research hotspot. In this study, the arterial blood of patients with hypertensive cerebral hemorrhage was collected and the blood clot model *in vitro* was prepared, and different doses of rt-PA intervention were given to observe the relationship between rt-PA and dissolution effect. This study aimed to provide theoretical support to further optimize the treatment of hypertensive intracerebral hemorrhage.

## Materials and Methods

### Patients

Thirty-five patients with hypertensive cerebral hemorrhage diagnosed from 2020-2-6 to 2020-6-30 in the Department of Neurosurgery of Rudong Hospital Affiliated with Nantong University and Haimen District People's Hospital were selected as the research objects. Inclusion criteria: (1) meet the diagnostic criteria for hypertensive intracerebral hemorrhage; (2) the hematoma was located in the basal ganglia, thalamus, or brain lobe; (3) consciousness disorder, hemiplegia, aphasia, limb numbness, and other neurological disorders; (4) the time from onset to hospitalization did not exceed 48 h; (5) normal cardiopulmonary function, normal blood coagulation function. Exclusion criteria: (1) age <18 years; (2) cerebral hemorrhage caused by other causes; (3) those with a history of surgery within one month; (4) abnormal blood coagulation function; (5) The time from onset to hospitalization exceeded 48 h; (6) oral anticoagulants; (7) drug users; (8) pregnant women; (9) severe heart, lung, kidney, and liver function abnormalities. Among the 35 patients with hypertensive intracerebral hemorrhage, 27 were males and 8 were females, ranging in age from 28 to 81 years old, with an average of 64.14 ± 13.00 years old. The average systolic blood pressure at admission was 171.34 ± 25.24 mmHg, and the average diastolic blood pressure was 96.91 ± 11.66 mmHg. There were 5 cases with a history of stroke; 3 cases with a history of diabetes; 13 cases with a history of hypertension were clearly stated, only 4 cases with a history of regular use of antihypertensive drugs; 2 cases with a history of smoking; 4 cases with a history of drinking. The studies involving human participants were reviewed and approved by the Ethics Committee of Rudong County People's Hospital and Haimen District People's Hospital. The patient's family provided written informed consent to participate in this study.

### Samples and Model

A total of 40 ml of arterial blood was drawn from each patient and randomly injected into 10 blood collection tubes (4 ml/tube), centrifuged, then the serum was aspirated to prepare the blood clot model, and divided into the standard group, control group, 0.125 mg/3ml, 0.25 mg/3ml, 0.5 mg/3ml, 1 mg/3ml, 2 mg/3ml, 3 mg/3ml rt-PA groups, 20,000 U, and 40,000 U urokinase (u-PA) groups. No intervention in the standard group for checking blood clot volume, the control group was added with 3 ml saline, then 0.125 mg/3 ml, 0.25 mg/3 ml, 0.5 mg/3 ml, 1 mg/3 ml, 2 mg/3 ml, 3 mg/3 ml rt-PA groups were added with the corresponding dose of the rt-PA (Boehringer-Ingelheim, Germany), and the 20,000 and 40,000 U u-PA groups were added with the corresponding dose of the u-PA (NDPHARM, China). Then, the tube was placed in a 37°C electric-heated thermostatic water bath (Wuxi Yierda, China), and the corresponding dissolved volume or blood clot volume was measured 30, 60, 90, 120, and 150 min after the intervention with a pipette gun (Eppendorf, Germany).

### Statistical Analysis

The SPSS 21.0 data software (IBM, NY, USA) package was used for the statistical analysis of the data obtained in this study. The measurement data obtained in the study was verified by the Shapiro-Wilk test to conform to the normal distribution, expressed by mean±SD, and the independent-test or ANOVA was used for comparison between groups. The relationship between blood clot volume and the blood test indexes of patients was analyzed with Spearman correlation analysis. *P* < 0.05 was considered the difference to be statistically significant.

## Results

### The Relationship Between Blood Clots of the Standard Group and Clinical Data

The blood clot volume of males was bigger than that of females at 0, 30, and 90 min (*P* < 0.05), and the difference was not statistically significant at 120 min, 150 min. There was no statistically significant difference between the subgroups of other clinical factors at any time point. The results were shown in Table 1.

**Table 1:** The relationship between blood clot of standard group and clinical data.

	**n**	**0 min**	**30 min**	**60 min**	**90 min**	**120 min**	**150 min**
Gender							
Male	27	1.53 ± 0.16	1.34 ± 0.18	1.25 ± 0.22	1.21 ± 0.23	1.17 ± 0.24	1.17 ± 0.24
Female	8	1.36 ± 0.16	1.15 ± 0.13	1.08 ± 0.13	1.03 ± 0.12	1.01 ± 0.12	1.01 ± 0.12
t		2.59	2.73	2.14	2.12	1.87	1.87
*P*		***0.01***	***0.01***	***0.04***	***0.04***	0.07	0.07
Age							
≥60	25	1.51 ± 0.17	1.30 ± 0.19	1.21 ± 0.23	1.18 ± 0.24	1.14 ± 0.24	1.14 ± 0.24
<60	10	1.45 ± 0.20	1.28 ± 0.18	1.20 ± 0.18	1.14 ± 0.18	1.11 ± 0.17	1.11 ± 0.17
*t*		0.84	0.37	0.14	0.50	0.30	0.30
*P*		0.41	0.72	0.89	0.62	0.77	0.77
History of stroke							
No	30	1.51 ± 0.18	1.31 ± 0.20	1.21 ± 0.23	1.17 ± 0.24	1.13 ± 0.24	1.13 ± 0.24
Yes	5	1.37 ± 0.09	1.25 ± 0.07	1.21 ± 0.08	1.16 ± 0.08	1.13 ± 0.08	1.13 ± 0.08
*t*		1.71	0.65	0.05	0.05	0.06	0.06
*P*		0.10	0.52	0.97	0.96	0.95	0.95
Medication history							
No	21	1.49 ± 0.17	1.30 ± 0.18	1.21 ± 0.22	1.16 ± 0.23	1.13 ± 0.25	1.13 ± 0.25
Yes	14	1.49 ± 0.19	1.29 ± 0.21	1.21 ± 0.21	1.17 ± 0.21	1.14 ± 0.20	1.14 ± 0.20
*t*		0.05	0.29	0.00	0.09	0.14	0.14
*P*		0.96	0.77	1.00	0.93	0.89	0.89
Smoking history							
No	33	1.48 ± 0.18	1.29 ± 0.19	1.20 ± 0.21	1.15 ± 0.22	1.12 ± 0.22	1.12 ± 0.22
Yes	2	1.65 ± 0.08	1.47 ± 0.04	1.42 ± 0.01	1.39 ± 0.02	1.36 ± 0.03	1.36 ± 0.03
*t*		1.27	1.37	1.42	1.47	1.51	1.51
*P*		0.21	0.18	0.17	0.15	0.14	0.14
Drinking history							
No	31	1.49 ± 0.18	1.29 ± 0.19	1.20 ± 0.22	1.16 ± 0.22	1.12 ± 0.23	1.12 ± 0.23
Yes	4	1.47 ± 0.22	1.31 ± 0.20	1.27 ± 0.19	1.24 ± 0.18	1.20 ± 0.21	1.20 ± 0.21
*t*		0.23	0.18	0.59	0.73	0.62	0.62
*P*		0.82	0.86	0.56	0.47	0.54	0.54
History of diabetes							
No		1.50 ± 0.18	1.31 ± 0.18	1.23 ± 0.21	1.18 ± 0.22	1.15 ± 0.23	1.15 ± 0.23
Yes		1.43 ± 0.04	1.11 ± 0.16	1.03 ± 0.18	0.99 ± 0.17	0.97 ± 0.17	0.97 ± 0.17
*t*		0.59	1.91	1.53	1.48	1.29	1.29
*P*		0.56	0.07	0.14	0.15	0.21	0.21

*Bold and italic values indicate P < 0.05*.

Hematocrit (HCT) was correlated with blood clot volume and the correlation decreased with time (*P* < 0.05). There was no correlation between other relevant blood test indexes and blood clot volume (*P* > 0.05). The results were shown in Table 2.

**Table 2:** The relationship between blood clot of standard group and blood test indexes.

	**0 min**	**30 min**	**60 min**	**90 min**	**120 min**	**150 min**
WBC (10∧9/L)	0.074	0.195	0.192	0.18	0.149	0.149
Neu (10∧9/L)	0.063	0.169	0.156	0.146	0.111	0.111
PT(s)	−0.037	0.075	0.119	0.14	0.146	0.146
INR	0.09	0.021	−0.062	−0.047	−0.092	−0.092
PLC (10∧9/L)	−0.066	−0.023	−0.009	0.01	0.017	0.017
FG (g/L)	−0.238	−0.213	−0.182	−0.206	−0.166	−0.166
DD (ug/L)	0.063	−0.061	−0.11	−0.134	−0.132	−0.132
FDP (ug/ml)	0.152	−0.107	−0.213	−0.214	−0.221	−0.221
HCT (%)	0.648^**^	0.628^**^	0.493^**^	0.488^**^	0.453^**^	0.453^**^

*DD, D-dimer; FDP, fibrinogen degradation product; FG, Fibrinogen; HCT, hematocrit; INR, international standardized ratios; Neu, neutrophil count; PLC, platelet count; PT, prothrombin time; WBC, white blood cell count*.

** *P < 0.01*.

### Effect of Time on the Hemolytic Efficiency of Drugs

**Figure 1:**
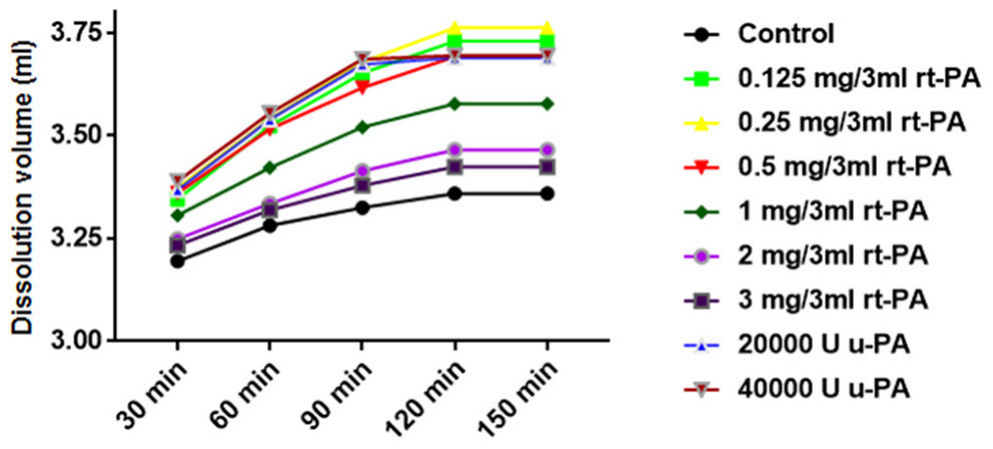
Effect of time or concentration on the hemolytic efficiency of drugs. The 30, 60, and 90 min dissolution curves of each group showed an upward trend (*P* < 0.05), and the dissolution curves tended to be flat at 120 and 150 min. The dissolution volume of 0.125 mg/3 ml, 0.25 mg/3 ml, 0.5 mg/3 ml rt-PA, 20,000 U, 40,000 U u-PA was higher than that of 1 mg/3 ml, 2 mg/3 ml, 3 mg/3 ml rt-PA volume (*P* < 0.05).

The dissolution volume of each group gradually increased at 30, 60, and 90 min (*P* < 0.05). The dissolution volume at 120 and 150 min in the 20,000 and 40,000 U u-PA groups was similar to that at 90 min. Although the dissolution volume of the other groups continued to increase at 120 min, there was no statistically significant difference compared with 90 min (*P* > 0.05). The dissolution volume at 150 min in all groups was similar to that at 120 min (Figure 1).

### Effect of Concentration on the Hemolytic Efficiency of Drugs

The dissolution volume of different doses of rt-PA and u-PA was higher than that of the control group (*P* < 0.05). Among them, the dissolution volume of 0.125 mg/3 ml, 0.25 mg/3ml, and.5 mg/3 ml rt-PA was not significantly different from that of 20,000 and 40,000 U u-PA (*P* > 0.05). The dissolution volume of.25 mg/3 ml rt-PA was slightly higher than that of.125 mg/3 ml and.5 mg/3 ml rt-PA, but the difference was not statistically significant (*P* > 0.05). The dissolution volume of.125 mg/3 ml, 0.25 mg/3 ml, 0.5 mg/3 ml rt-PA, 20,000 U, 40,000 U u-PA was higher than that of 1 mg/3 ml, 2 mg/3 ml, and 3 mg/3 ml rt-PA (*P* < 0.05). The dissolution volume of 1 mg/ml rt-PA was higher than the dissolution volume of 2 mg/3 ml and 3 mg/3 ml rt-PA (*P* < 0.05). There was no significant difference in the dissolution volume of 2 mg/3 ml and 3 mg/3 ml rt-PA (*P* > 0.05) (Figure 1; Table 3). Gender differences had no effect on the effects of the above drugs (Table 4).

**Table 3:** Effect of drugs concentration on the hemolytic efficiency.

**Group**	**30 min**	**60 min**	**90 min**	**120 min**	**150 min**
Control	3.20 ± 0.15	3.28 ± 0.21	3.32 ± 0.21	3.36 ± 0.22	3.36 ± 0.22
0.125 mg/3 ml rt-PA	3.35 ± 0.20^*^	3.52 ± 0.26^*^	3.65 ± 0.29^*^	3.73 ± 0.30^*^	3.73 ± 0.30^*^
0.25 mg/3 ml rt-PA	3.37 ± 0.20^*^	3.54 ± 0.26^*^	3.68 ± 0.28^*^	3.76 ± 0.30^*^	3.76 ± 0.30^*^
0.5 mg/3 ml rt-PA	3.36 ± 0.24^*^	3.52 ± 0.27^*^	3.62 ± 0.28^*^	3.69 ± 0.28^*^	3.69 ± 0.28^*^
1 mg/3 ml rt-PA	3.31 ± 0.21^*@^	3.42 ± 0.23^*@^	3.52 ± 0.26^*@^	3.58 ± 0.26^*@^	3.58 ± 0.26^*@^
2 mg/3 ml rt-PA	3.25 ± 0.18^*@#^	3.34 ± 0.19^*@#^	3.41 ± 0.23^*@#^	3.47 ± 0.22^*@#^	3.47 ± 0.22^*@#^
3 mg/3 ml rt-PA	3.23 ± 0.18^*@#^	3.32 ± 0.18^*@#^	3.38 ± 0.19^*@#^	3.42 ± 0.18^*@#^	3.42 ± 0.18^*@#^
20,000 U u-PA	3.37 ± 0.24^*@#∧^	3.54 ± 0.26^*@#∧^	3.67 ± 0.29^*#∧^	3.69 ± 0.30^*#∧^	3.69 ± 0.30^*#∧^
40,000 U u-PA	3.39 ± 0.26^*#∧^	3.55 ± 0.28^*#∧^	3.69 ± 0.30^*#∧^	3.70 ± 0.30^*#∧^	3.70 ± 0.30^*#∧^

*rt-PA, alteplase; u-PA, urokinase*.

* *VS. Control group, P < 0.05*;

@ *VS. 0.125 mg/3ml, 0.25 mg/3 ml, and 0.5 mg/3 ml rt-PA groups, P < 0.05*;

# *VS. 1 mg/3 ml rt-PA group, P < 0.05*;

∧ *VS. 2 mg/3 ml, and 3 mg/3 ml rt-PA groups, P < 0.05*.

**Table 4:** Effect of drugs concentration on the hemolytic efficiency between male and female.

**Group**	**Gender**	**30 min**	**60 min**	**90 min**	**120 min**	**150 min**
Control	Male	3.19 ± 0.14	3.28 ± 0.21	3.32 ± 0.22	3.36 ± 0.23	3.36 ± 0.23
	Femal	3.21 ± 0.17	3.29 ± 0.20	3.33 ± 0.20	3.35 ± 0.20	3.35 ± 0.20
0.125 mg/3ml rt-PA	Male	3.33 ± 0.21	3.51 ± 0.28	3.64 ± 0.31	3.72 ± 0.32	3.72 ± 0.32
	Femal	3.39 ± 0.18	3.56 ± 0.20	3.68 ± 0.22	3.76 ± 0.22	3.76 ± 0.22
0.25 mg/3ml rt-PA	Male	3.37 ± 0.21	3.53 ± 0.27	3.68 ± 0.30	3.77 ± 0.32	3.77 ± 0.32
	Femal	3.40 ± 0.18	3.57 ± 0.20	3.68 ± 0.20	3.75 ± 0.21	3.75 ± 0.21
0.5 mg/3ml rt-PA	Male	3.35 ± 0.25	3.51 ± 0.28	3.62 ± 0.30	3.69 ± 0.30	3.69 ± 0.30
	Femal	3.39 ± 0.21	3.53 ± 0.24	3.62 ± 0.23	3.69 ± 0.23	3.69 ± 0.23
1 mg/3ml rt-PA	Male	3.30 ± 0.22	3.42 ± 0.24	3.52 ± 0.27	3.58 ± 0.27	3.58 ± 0.27
	Femal	3.32 ± 0.20	3.43 ± 0.22	3.51 ± 0.24	3.57 ± 0.23	3.57 ± 0.23
2 mg/3ml rt-PA	Male	3.24 ± 0.19	3.33 ± 0.18	3.41 ± 0.23	3.47 ± 0.23	3.47 ± 0.23
	Femal	3.27 ± 0.18	3.35 ± 0.21	3.41 ± 0.21	3.45 ± 0.22	3.45 ± 0.22
3 mg/3ml rt-PA	Male	3.23 ± 0.18	3.31 ± 0.18	3.38 ± 0.19	3.43 ± 0.18	3.43 ± 0.18
	Femal	3.25 ± 0.18	3.33 ± 0.20	3.38 ± 0.20	3.41 ± 0.20	3.41 ± 0.20
20,000 U u-PA	Male	3.36 ± 0.25	3.54 ± 0.28	3.68 ± 0.30	3.69 ± 0.31	3.69 ± 0.31
	Femal	3.39 ± 0.19	3.55 ± 0.23	3.66 ± 0.24	3.68 ± 0.26	3.68 ± 0.26
40,000 U u-PA	Male	3.38 ± 0.27	3.55 ± 0.29	3.69 ± 0.31	3.70 ± 0.31	3.70 ± 0.31
	Femal	3.41 ± 0.20	3.57 ± 0.25	3.68 ± 0.26	3.69 ± 0.27	3.69 ± 0.27

*rt-PA, alteplase; u-PA, urokinase*.

## Discussion

In this study, the arterial blood of patients with hypertensive intracerebral hemorrhage was used to create a blood clot model *in vitro* to explore the effect of fibrinolytic drugs on the hematoma. Because hypertensive cerebral hemorrhage intracranial hematoma is formed by arterial blood coagulation after arterial rupture, the experimental blood clot model was closer to the clinic, and the results were more credible. The method of constructing the blood clot model was scientific and reliable (13). In addition, we found that due to the separation effect of serum, the rt-PA and u-PA solutions could not interact with blood clots in the preliminary experiment, after the serum was separated by centrifugation, the experiment proceeded smoothly.

The results showed that the concentration of rt-PA was critical to the dissolution effect, and the dissolution efficiency of rt-PA on arterial blood clots was negatively correlated with the drug concentration. The dissolution efficiency of u-PA or rt-PA was the best in the 30–90 min after the intervention, and the plateau period was obvious after 90 min. U-PA and rt-PA were added to arterial blood clots *in vitro*, and the dissolution efficiency of u-PA was the best at 90 min, the dissolution efficiency of rt-PA was slightly lower than that of u-PA. However, u-PA is more likely to cause bleeding when used in the body, and it is prone to pollution during production. It has been banned by the US Food and Drug Administration (14), so rt-PA was studied in this study. The results also showed that the dissolution efficiency of low-concentrations rt-PA (0.125, 0.25, and 0.5 mg groups) was higher than that of the high-concentration group (1, 2, and 3 mg groups), which was close to or reached the dissolution efficiency of the u-PA groups, and there was no statistically significant difference between the low concentration groups. The results suggest that the dissolution efficiency of rt-PA does not have a positive correlation with the concentration, which may be related to the enzymatic reaction, that is, when the substrate concentration is constant, the increase in the enzyme concentration is not positively related to the reaction speed, and even inhibition occurs. Clinical related reports also pointed out that when the concentration of rt-PA was too high, it was easy to induce neuroinflammation and aggravate edema (9, 13, 15, 16). Therefore, we speculate that low concentrations of rt-PA may be safer and more effective in clinical applications. It is not recommended to use rt-PA with a concentration higher than.5 mg/3 ml for the treatment of intracranial hematoma. Since our experiment was an *in vitro* experiment, it cannot completely simulate the intracranial environment. The optimal concentration needs to be confirmed by further animal models or human clinical experiments.

The results of this experiment also observed that time were an important factor for the dissolution effect. Ninety min after adding the rt-PA, the dissolution effect of the rt-PA increased with time, showing a positive correlation, and the slope of the dissolution curve was large; after 90 min, the slope of the dissolution curve tended to be flat; after 120 min, there was no change in the slope of the dissolution curve. It is inferred that 90–120 min after adding the medicine is the ideal time to drain the liquefied hematoma, and the clamping time of the drainage tube should not exceed 120 min in the clinical application of rt-PA for intracranial hematoma. However, our data were the results of *in vitro* experiments and the related animal experiment or should clinical experiments be explored in the following days; it should be determined whether to add rt-PA in time based on the residual situation of the hematoma examined by the head CT and the improvement of clinical symptoms; the sample size of patients is small, especially the sample size of female patients is small, but this is roughly the same as the ratio of male to female patients with hypertensive intracerebral hemorrhage in China. The results of gender subgroup analysis showed that gender differences had no effect on the effects of the above drugs.

In summary, *in vitro*, low-concentration rt-PA has a better dissolution effect, and it shows a time-dependent effect, reaching the highest effect in 90 min, which maybe provides theoretical support to further optimize the treatment of hypertensive intracerebral hemorrhage.

## Data Availability Statement

The raw data supporting the conclusions of this article will be made available by the authors, without undue reservation.

## Ethics Statement

The studies involving human participants were reviewed and approved by Ethics Committee of Rudong County People's Hospital and Haimen District People's Hospital. The patients/participants provided their written informed consent to participate in this study. Written informed consent was obtained from the individual(s) for the publication of any potentially identifiable images or data included in this article.

## Author Contributions

All authors listed have made a substantial, direct, and intellectual contribution to the work and approved it for publication.

## Funding

This work was supported by the Jiangsu Provincial Science and Technology Program (BRA2019206) and the fifth phase of Project 333 scientific research in Jiangsu Province in 2019 (BRA2019206).

## Conflict of Interest

The authors declare that the research was conducted in the absence of any commercial or financial relationships that could be construed as a potential conflict of interest.

## Publisher's Note

All claims expressed in this article are solely those of the authors and do not necessarily represent those of their affiliated organizations, or those of the publisher, the editors and the reviewers. Any product that may be evaluated in this article, or claim that may be made by its manufacturer, is not guaranteed or endorsed by the publisher.
